# Enhanced detection of subtle cortical abnormalities in focal epilepsy using 7 T MRI surface-based models and graph neural networks

**DOI:** 10.1007/s00234-026-04103-8

**Published:** 2026-07-06

**Authors:** Matteo Lenge, Simona Fiori, Pietro Cappelletto, Andrea Droghini, Elisa Barbi, Anna Maria Buccoliero, Graziella Donatelli, Michela Tosetti, Flavio Giordano, Carmen Barba, Renzo Guerrini

**Affiliations:** 1 Neuroscience and Human Genetics Department, Meyer Children‘s Hospital IRCCS, Florence, Italy; 2 University of Florence, Florence, Italy; 3 IMAGO7 Foundation, Pisa, Italy; 4 Neuroradiology Unit, Department of Translational Research on New Technologies in Medicine and Surgery, University of Pisa, Pisa, Italy; 5 Laboratory of Medical Physics and Magnetic Resonance, IRCCS Fondazione Stella Maris, Pisa, Italy

**Keywords:** Focal epilepsy, Focal cortical dysplasia, MRI, Computational MRI, Artificial intelligence

## Abstract

**Purpose:**

MRI detection of subtle focal cortical dysplasia (FCD)-like abnormalities remains challenging in focal epilepsy. Higher signal-to-noise ratio and spatial resolution offered by ultra-high-field 7T MRI and surface-based graph-neural-network (GNN) analysis may improve detection of subtle cortical abnormalities. We evaluated whether combining 7T MRI with a surface-based GNN classifier improves lesion detection in focal epilepsy of suspected structural origin.

**Methods:**

We analyzed paired 7T and 3T MRI datasets from 87 patients with focal epilepsy (78.1% pediatric) and 10 internal healthy control individuals. We processed T1-weighted and Fluid-Attenuated-Inversion-Recovery MRI using a surface-based framework and a pre-trained GNN classifier developed within the Multi-centre-Epilepsy-Lesion-Detection project. We compared classifier outputs with expert visual MRI assessment, clinical and surface electroencephalography (EEG) localization (all patients), stereo-EEG (ten patients) and histopathological (17 patients) findings. We evaluated diagnostic yield and lesion conspicuity, and performed within-subject comparisons between 7T and 3T.

**Results:**

Following quality controls, we included 70 patients. The 7T MRI-based classifier identified lesion clusters concordant with visual 3T MRI and electroclinical localization in 25/37 (67.6%) MRI-positive patients, electroclinical-concordant clusters in 15/33 (45.4%) 3T MRI-negatives, stereo-EEG-concordant clusters in 7/10 (70.0%) patients and surgically-concordant clusters in 11/17 (64.7%). Among classifier-positive patients (40/70, 57.1%), 7T allowed detection of previously hidden lesions in 15/40 (37.5%) patients, and improved detection of known lesions in 11/40 (27.5%).

**Conclusion:**

Combining 7T MRI with surface-based GNN analysis improves detection and characterization of FCD-like abnormalities in focal epilepsy, particularly in patients with unrevealing 3T MRI, supporting the adoption of advanced neuroimaging in presurgical epilepsy assessment.

**Supplementary Information:**

The online version contains supplementary material available at https://doi.org/10.1007/s00234-026-04103-8.

## Introduction

Focal cortical dysplasia (FCD) is the most frequent etiology of drug-resistant focal epilepsy in children, and an identifiable structural MRI lesion is one of the most significant predictors of a positive outcome if surgical treatment of epilepsy is planned [1]. In children, surgery for epilepsy can achieve 80% seizure-free outcome after completely removal of the epileptogenic MRI lesion [2]. Accurate identification and characterization of the epileptogenic lesion is therefore essential [3]. Conversely, if structural MRI abnormalities remain ill-defined and resection incomplete, only 20–50% of patients achieve seizure freedom [4]. An unrevealing brain MRI further reduces the likelihood of seizure control and may require more thorough presurgical evaluation, including functional MRI, fluorodeoxyglucose - positron emission tomography (FDG-PET) and invasive electroencephalography (EEG) investigations. Large multicenter studies show that, for patients with normal MRI and unrevealing or non-specific histopathology, post-surgery seizure freedom is about 40% at two years, with even poorer outcomes in extratemporal epilepsy [5]. In pediatric cohorts, seizure-free rates after surgery for epilepsy with unrevealing MRI are around 25%, and strongly influenced by clinical factors such age at onset, frequency of seizures, and number of previously failed antiseizure medications [6].

Although invasive intracranial procedures, particularly stereo-EEG, can help identifying the seizure onset zone (SOZ) [7], their use remains selective, has important limitations, and is associated with risks of hemorrhage, infection, and neurological deficits [8]. Large series of surgical procedures show that most MRI-negative patients undergo invasive investigations, but only a minority achieve long-term seizure freedom [9]. These considerations highlight the need for improved non-invasive methods that reduce dependence on invasive mapping.

In this context, conventional MRI has limitations related to its being qualitative, reader-dependent, and prone to overlooking subtle structural abnormalities that may underlie epileptogenic networks [10]. Recent developments focused on improving the detection of subtle epileptogenic abnormalities through advances in neuroimaging and computational analysis [1]. 3 T MRI offers improved diagnostic yield (+ 5%) compared with lower-field scanners [11] and high-resolution 7 T MRI accurately visualises FCD [12], enabling an unprecedented understanding of the laminar organization of the cortex [13], and substantially increasing (8% − 67%) the number of lesions identified [14–20].

Further improvements have been provided by advanced post-processing methods, including automated voxel-based [21, 22] or post-processing techniques [23, 24], also supported by machine-learning approaches [25, 26]. Advanced computational-based detectors have significantly improved sensitivity and specificity [23, 27], up to uncovering subtle gradients of severity from dysplastic to normal cortex [28]. A surface-based classifier using 33 features and artificial neural networks demonstrated 59% sensitivity and 54% specificity in a large multicenter dataset of 1015 participants [29]. More recently, integrating graph neural networks (GNN) in the pipeline has yielded substantially higher performances increasing sensitivity to 81.6%, while preserving interpretability and facilitating clinical adoption [30]. These multicenter, open-access initiatives have produced robust and explainable algorithms that support clinicians in identifying subtle cortical abnormalities in focal epilepsy.

We adapted the surface-based GNN classifier developed by Ripart and colleagues [30] to 7 T structural MRI scans of patients with focal epilepsy and visible or suspected structural etiology on conventional 3 T imaging, and tested our approach on 7 T of visible lesions and evaluated performance on suspected abnormalities. In our approach, we evaluated classifier outputs at 7 T against expert MRI visual assessment, electroclinical SOZ localization, and available intracranial stereo-EEG and neuropathological findings. We also performed within-subject comparisons between 7 T and 3 T MRI to assess the added value of ultra-high field imaging on classifier performance and lesion detectability/characterization. We hypothesized that combining 7 T MRI with surface-based machine learning analysis would better identify subtle cortical abnormalities beyond conventional structural MRI and outperform equivalent analyses conducted on 3 T data.

## Materials and methods

### Study design

We designed a prospective cohort study to evaluate the performance of the surface-based GNN algorithm developed within the MELD project [30] for the detection of cortical abnormalities using 7 T brain MRI. Structural 7 T brain MRI scans were acquired between 2015 and 2024 at a multicenter consortium. We recruited a control group from the community as part of the research protocol. The study was approved by the Pediatric Ethics Committee of the Tuscany Region, Italy (Ethics approval number 212/2024). Written informed consent for research use of imaging data was obtained from all participants or parents/legal guardians in the case of minors.

### Participants and clinical data

We enrolled eligible participants at the tertiary pediatric neurology center of Meyer Children’s Hospital IRCCS, Florence, Italy. Inclusion criteria were: (1) age between 8 and 65 years; (2) focal epilepsy; (3) clinical and EEG findings consistent with a focal SOZ; and (4) at least one prior 3 T brain MRI examination. Exclusion criteria were: (1) contraindication to MRI; and (2) inability to cooperate during awake MRI acquisition.

We collected clinical, electrophysiological and neuroimaging data from routine clinical care and presurgical evaluations for the purpose of localizing the SOZ and establishing a reference for comparison with automated classifier outputs. These data included detailed seizure semiology, long-term video-EEG monitoring, and expert review of structural 3 T MRI. When available as part of the presurgical evaluation, we also collected additional investigations including intracranial stereo-EEG, FDG-PET imaging, and neuropsychological assessment. For participants who underwent epilepsy surgery and whose histopathological findings were available, we classified FCD according to the 2022 International League Against Epilepsy (ILAE) consensus classification method [31].

We retrieved brain MRI data from the most recent clinical 3 T epilepsy imaging protocol for all patients. Examinations at 3 T were performed on two scanners equipped with 8-channels head coils (Achieva, Philips Healthcare, The Netherlands; Excite HDx, GE Medical Systems, Milwaukee, WI). The 3 T protocol included whole-brain 3D T1-weighted (T1w) fast-spoiled gradient echo (FSPGR), whole-brain 3D T2w Fluid Attenuation Inversion Recovery (3D-FLAIR), 2D T2w Fast Spin-Echo, T2*w Gradient-echo (GRE), and 2D T1w sequences. We reviewed all 3 T MRI scans on a dedicated workstation using the Osirix MD by expert neurologists, neuroradiologists and neurosurgeons with longstanding experience in epilepsy neuroimaging. Reviewers were provided with clinical and EEG information indicating the suspected SOZ. We examined all available multimodal dataset, including spatially co-registered FDG-PET imaging, post-stereo-EEG implantation MR/CT imaging, and postsurgical MRI, using 3D Slicer software [32]. Image interpretation was performed by consensus, with particular attention to the identification of structural abnormalities and their concordance with electroclinical data.

Based on the combined assessment of 3 T MRI findings and electroclinical data, we classified patients with univocal concordant interictal, ictal and seizure-onset localization to a single cortical region into two subgroups: 3 T MRI-positive, defined by evidence for a structural abnormality consistent with focal cortical dysplasia (FCD) or a different type of epileptogenic lesion; 3 T MRI-negative, defined as absence of detectable lesions at 3T.

To evaluate concordance between visual 3 T MRI assessment and automatically generated lesion clusters, we mapped the visually identified lesion in 3 T MRI-positive subjects onto the FreeSurfer labeling system [33]. In 3 T MRI-negative subjects, we used all available imaging and electroclinical data to define the SOZ, which was likewise mapped onto the FreeSurfer labeling system [33].

###  7 T images acquisition and visual assessment

Participants underwent 7 T MRI (GE Healthcare, USA) equipped with a 2-channel transmit/32-channel receive head coil (Nova Medical, USA). The imaging protocol included: 3D T1w magnetization-prepared rapid gradient-echo (MP-RAGE) (inversion time [TI] 1100 ms, flip angle 8°, matrix 276 × 276, slice thickness 0.80 mm, field-of-view (FOV) 22 cm, voxel resolution 0.8 × 0.8 × 0.8 mm); 3D sagittal T2w CUBE FLAIR (time repetition [TR]/time echo [TE] 10000/123 ms, matrix 320 × 320, slice thickness 0.70 mm, field-of-view (FOV) 22 cm, voxel resolution 0.7 × 0.7 × 0.7 mm isotropic); 2D T2w fast spin-echo XL (FSE-XL) (time repetition [TR]/time echo [TE] 7000/60 ms, flip angle 142°, matrix 640 × 640, slice thickness 2.0 mm, field-of-view (FOV) 20 cm, voxel resolution 0.31 × 0.31 × 2.0 mm); 3D susceptibility-weighted angiography (SWAN) (time repetition [TR]/time echo [TE] 54.1/minimum ms, flip angle 15°, matrix 512 × 384, slice thickness 1.2 mm, field-of-view (FOV) 16.8 cm, voxel resolution 0.33 × 0.44 × 1.2 mm); 2D axial gradient-echo (GRE) dual-echo high-resolution (time repetition [TR]/time echo [TE] 500/10–20 ms, flip angle 30°, matrix 448 × 448, slice thickness 2.0 mm, field-of-view (FOV) 11.2 cm, voxel resolution 0.25 × 0.25 × 2.0 mm). 3D T1w and 3D FLAIR sequences were acquired at a whole brain level for surface-based analyses.

We visually reviewed all MRI datasets for structural abnormalities and artifacts or quality issues that could compromise subsequent computational analyses.

###  7 T and 3 T MRI surface-based cortical reconstruction

For post-processing of 7 T MRI data, we compensated transmit and receive field inhomogeneities by applying intensity-bias correction implemented in the unified segmentation pipeline [34] of SPM12 (MATLAB R2023b), with customized parameters (FWHM = 18 mm, sampling distance = 2 mm) [35]. We performed cortical surface reconstruction from sub-millimetric 3D-T1w images using the FreeSurfer high resolution pipeline (https://surfer.nmr.mgh.harvard.edu/fswiki/HiResRecon*)* [36]. Processing steps included motion correction, intensity normalization, skull stripping, and reconstruction of the white/grey matter interface (*white* surface) and the gray matter/cerebrospinal fluid (CSF) interface (*pial* surface). Since gray matter intensity was reduced in the temporal lobes due to altered local transmission efficiency, we applied custom parameters to the *mris_make_surfaces* command [36]. For *pial surface* refinement, we extended the processing by incorporating the steps associated with the *-FLAIRpial* option of the *recon-all* command (https://surfer.nmr.mgh.harvard.edu/fswiki/ReconAllDevTable*).* These steps included: registration of the 3D-FLAIR image to the 3D-T1w using *bbregister*, intensity normalization of the 3D-FLAIR (*mri_normalize*); and triangular tessellation [37], to obtain the final *pial surface* representation (*mris_make_surfaces*).

For the 3 T MRI data, we processed 3D-T1w and 3D-FLAIR images using the standard FreeSurfer pipeline (version 7.2.0, https://surfer.nmr.mgh.harvard.edu*)* [38].

At each step of processing, we ensured output accuracy through visual inspection, and manual corrections when necessary. We adopted a quality control procedure to exclude datasets presenting segmentation errors or unrecoverable artefacts in cortical surface reconstruction.

###  7 T and 3 T MRI surface-based feature assessment

For lesion detection, we employed the GNN lesion classifier, originally developed and validated by Ripart and colleagues [30] within the MELD project (https://meldproject.github.io/*).* The entire processing pipeline is freely available (https://github.com/MELDProject/meld_graph*)* and provides a pretrained model for lesion detection in new patients. For each subject and for both 7 T and 3 T surface reconstructions, we computed the 11 morphological and intensity features required by the MELD classifier: (i) cortical thickness; (ii) grey-white matter contrast; (iii) mean curvature; (iv) sulcal depth; and (v) intrinsic curvature. We evaluated the FLAIR signal intensity at (vi) 25%, (vii) 50%, and (viii) 75% of cortical thickness, (ix) at the grey-white matter boundary, and (x) 0.5 and (xi) 1.0 mm below the grey-white matter boundary. We smoothed and registered all features to a bilaterally symmetrical template surface (*fs_average_sym*). To exclude individuals with outlier feature values, we adopted quality control procedures according to the MELD protocol detailed in Spitzer et al. [29].

We then harmonized the features using ComBat [39] to mitigate non-biological variability related to scanner type and acquisition protocols. We performed four distinct harmonization procedures: two for the 7 T system before and after hardware upgrades and two for the 3 T scanners. To describe the effect of ComBat on 7 T and 3 T MRI features, we assessed the probability density profiles for the feature distributions before and after harmonization.

Standardization was performed using the parameters of the pre-trained MELD model. According to the MELD pipeline [29, 30], we subsequently normalized the features using three complementary approaches: (i) intra-subject z-score normalization to account for interindividual variability; (ii) interhemispheric asymmetry normalization to highlight vertices deviating from the contralateral hemisphere; and (iii) inter-subject normalization to adjust for physiological regional variability.

### Single-subject lesion classification

For each patient and harmonization dataset, we applied the GNN pipeline (*new_pt_pipeline*) [29] and used a dedicated Python script to generate a final report summarizing the features of each predicted lesion cluster, including cluster location, size and, across normalization strategies, z-scored mean feature values and integrated gradients saliency scores.

The final report described each cluster in terms of its anatomical location overlaid on MRI, size, and laterality, and visualized feature abnormalities using three horizontal bars corresponding to ComBat-harmonized, normalized, and asymmetry-based features. The x-axis represented the z-score values derived from standardization against vertex-wise distributions obtained from healthy controls. Feature saliency was visualized using a red-blue color scale based on integrated gradients [40], where positive saliency values indicated features contributing to lesion classification and negative values reflected features opposing lesion detection.

### Within-subject concordance with clinical and electrophysiological data

For 3 T MRI-positive patients, we evaluated the spatial concordance between automated lesion clusters identified on 7 T and 3 T MRI and visually defined lesion masks based on the FreeSurfer labeling system [33]. In 3 T MRI-negative patients without imaging evidence of focal morpho-structural abnormalities, we mapped SOZ localization onto the same cortical surface atlas used for the FreeSurfer segmentation [33] to enable direct comparison with lesion predictors generated by the automated classifiers.

Finally, in patients undergoing intracranial stereo-EEG recordings, we assessed the correspondence between the electrophysiologically defined SOZ and 7T-derived lesion clusters following the procedure described by Wang et al. [22].

### Within-subject comparison of lesion classification between 7 T and 3 T MRI

We performed within-subject comparisons between the 7 T and 3 T pipelines to characterize classifier performance and evaluate lesion conspicuity in terms of feature significance, cluster saliency, spatial extent, and robustness.

For each patient, we extracted tabular outputs from the 7 T and 3 T pipelines describing the detected lesion and features associated with it, including statistical measures (z-scores, standard deviations) and predictive importance (saliency values). For conspicuity analyses, we included only patients where both 7 T and 3 T pipelines identified a concordant lesion cluster. We conducted statistical analyses separately for each cluster, feature and metric. We assessed normality of differences in z-scores and saliency values using the Shapiro-Wilk test. For normally distributed data, we applied paired-sample t-tests, but used the Wilcoxon signed-rank test for non-normally distributed data. To correct for multiple comparisons, we adjusted p-values using the Benjamini-Hochberg false discovery rate (FDR) method. We defined the statistical significance as *p* < 0.05.

## Results

### Demographics and clinical characteristics

We studied 87 patients (41 females, 46 males; mean age 16.3 years, median 13.0, range 7.0–47.0), and 10 control healthy individuals (mean age 14.2 years, median 13.4, range 3.4–27.0) exclusively used as independent and internal validation cohort to evaluate the specificity of the pipeline on our local 7 T data. Demographic and clinical characteristics of the enrolled participants are summarized in Supplementary Table 1.

###  7 T MRI surface-based cortical reconstruction and feature assessment

After visual inspection of the MRI sequences and surface-based reconstructions, we excluded 17 of the 87 (19.5%) 7 T MRI datasets (12 of the MRI-positive subgroup, five of the MRI-negative group; see Supplementary Table 1 for details). Exclusion criteria were movement artefacts in T1w (6/17 patients) or FLAIR (3/17 patients) MRI, MRI acquisition without whole-brain coverage (3/17 patients), and surface reconstruction error due to field inhomogeneities in 5/17 patients in whom either the *white* or *pial* surfaces were inaccurately segmented (Supplementary Fig. 1b). Since processing was performed at the whole-brain level, we excluded from quantitative analyses lesion clusters located within regions affected by segmentation inaccuracies. In three 3 T MRI-positive patients (Patients 3, 27, and 32), scans performed inaccurate segmentations adjacent to the visually identified lesion. In these individuals (Supplementary Figs. 1a and c), the 7 T detector correctly identified a lesion cluster, which was therefore considered as a positive detection result. Individuals’ post-quality-control inclusion/exclusion process is illustrated in the flowchart reported in Supplementary Fig. 2.

Analysis on feature distributions before and after harmonization with ComBat showed pre-harmonization differences between feature profiles at 7 T and 3 T (Supplementary Figs. 3–6). Following harmonization, the main distribution peaks largely overlapped, indicating an effective reduction of non-biological variabilities. At the same time, visual inspection of the post-harmonization profiles showed that specific distribution patterns maintained their original field-intensity-dependent characteristics. Specifically, for grey–white matter contrast (Supplementary Fig. 3a-d), after alignment of the distribution centers, the 7 T cohort retained a higher and narrower peak density than the 3 T cohort, reflecting the intrinsically higher tissue-contrast properties of ultra-high-field MRI. For cortical thickness (Supplementary Fig. 3e-h), the 7 T and 3 T distributions showed substantial overlap after correction of the initial leftward shift observed in the 7 T data, but the 3 T profile showed a higher peak density at the mode, as expected [41].

###  7 T MRI visual assessment

**Fig. 1:**
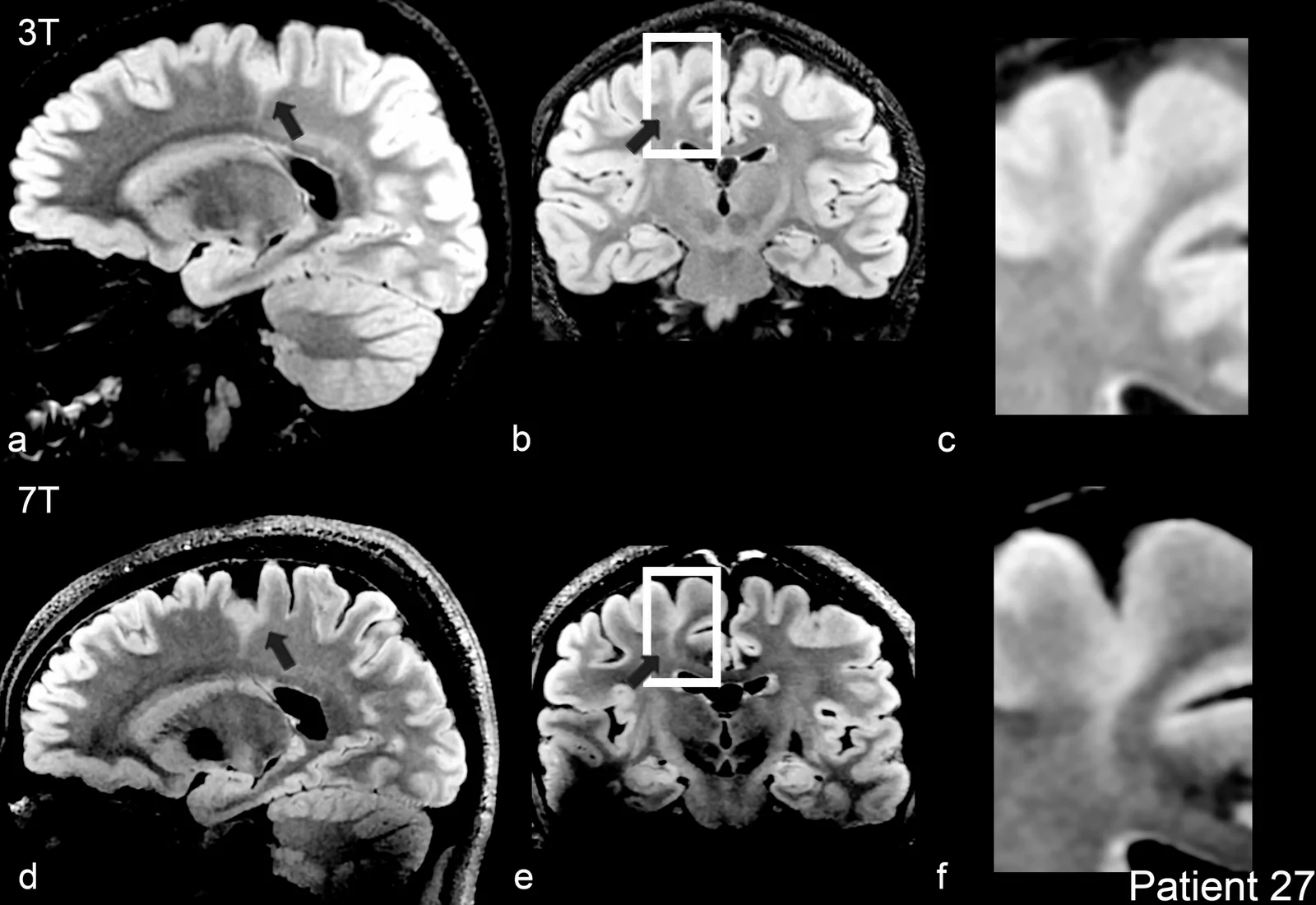
3 T and 7 T MRI in a patient with suspected focal cortical dysplasia (FCD) (Patient 27). Panels **a**-**c** show 3 T sagittal (**a**), coronal (**b**) and magnified coronal (**c**) FLAIR images, while **d-f** show the corresponding 7 T images. The images demonstrate a transmantle sign, suggestive of FCD-II, and magnified views (**c** and **f**) show improved delineation of the sulcus with loss of grey matter boundaries at 7 T MRI (**f**) compared with 3 T MRI (**c**)

The final quantitative cohort comprised 70 patients. Among these, 37/70 (52.9%) had a visible lesion MRI at 3 T (indicated in the following as ‘3T MRI-positive’ patients), which was confirmed by visual assessment at 7 T (Fig. 1). Of the remaining 33/70 patients (47.1%) with negative 3 T MRI (‘3T MRI-negative’ patients), visual analysis of 7 T MRI revealed subtle structural abnormalities in two (6.0%).

###  7 T surface-based lesion classification

**Fig. 2:**
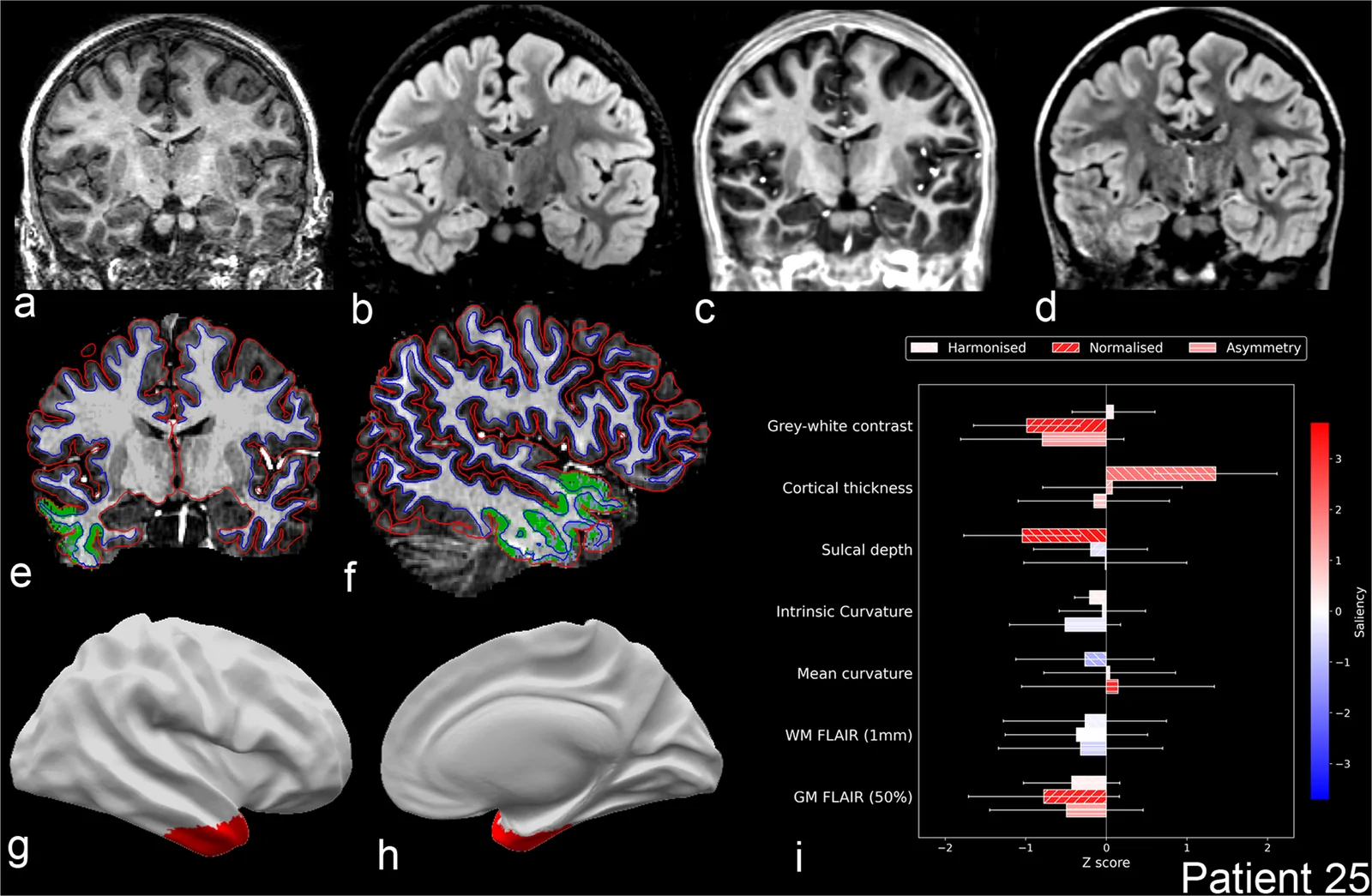
3 T and 7 T MRI, and 7 T segmentation and cortical surface-based reconstruction, and classifier results in a patient with a positive 3 T MRI (Patient 25). Coronal 3 T T1w (**a**) and FLAIR (**b**) MRI, and corresponding 7 T T1w (**c**) and FLAIR (**d**) MRI; coronal (**e**) and sagittal (**f**) 7 T T1w MRI after skull-stripping, with superimposed the lesion cluster detected by the 7 T classifier (green overlay) and *white* (blue line) and *pial* (red line) surfaces; 3D cortical surface models of the lateral (**g**) and mesial (**h**) right hemisphere with superimposed lesion cluster detected by the 7 T classifier (red overlay); 7 T classifier feature profile (**i**) of the lesion cluster

For all individuals studied (see an example of the pipeline output in Fig. 2), we obtained a quantitative representation of abnormal morphometric features associated with each detected lesion. As detailed in Supplementary Table 1, among the 40/70 patients who showed positive concordance with visual MRI, electroclinical localization, or both, the 7 T surface-based GNN pipeline identified a single cluster in 38/40 (95.0%) patients. In the remaining 2/40 patients, two clusters were identified (5.0% false-positive rate). Conversely, among the 30/70 patients without concordance between lesion detection output and visual MRI, electroclinical localization, or both, no cluster was detected in 25/30 (83.3%) patients. In the remaining 5/30 (16.7%) patients, the 7 T surface-based GNN pipeline detected a lesion cluster that was not concordant with visual MRI, electroclinical localization, or both (16.7% false-positive rate).

Application of the 7 T lesion classifier to the internal 7 T control cohort identified a lesion cluster in 1/10 controls (10% false-positive rate).

###  7 T classifier concordance with clinical and electrophysiological data

**Fig. 3:**
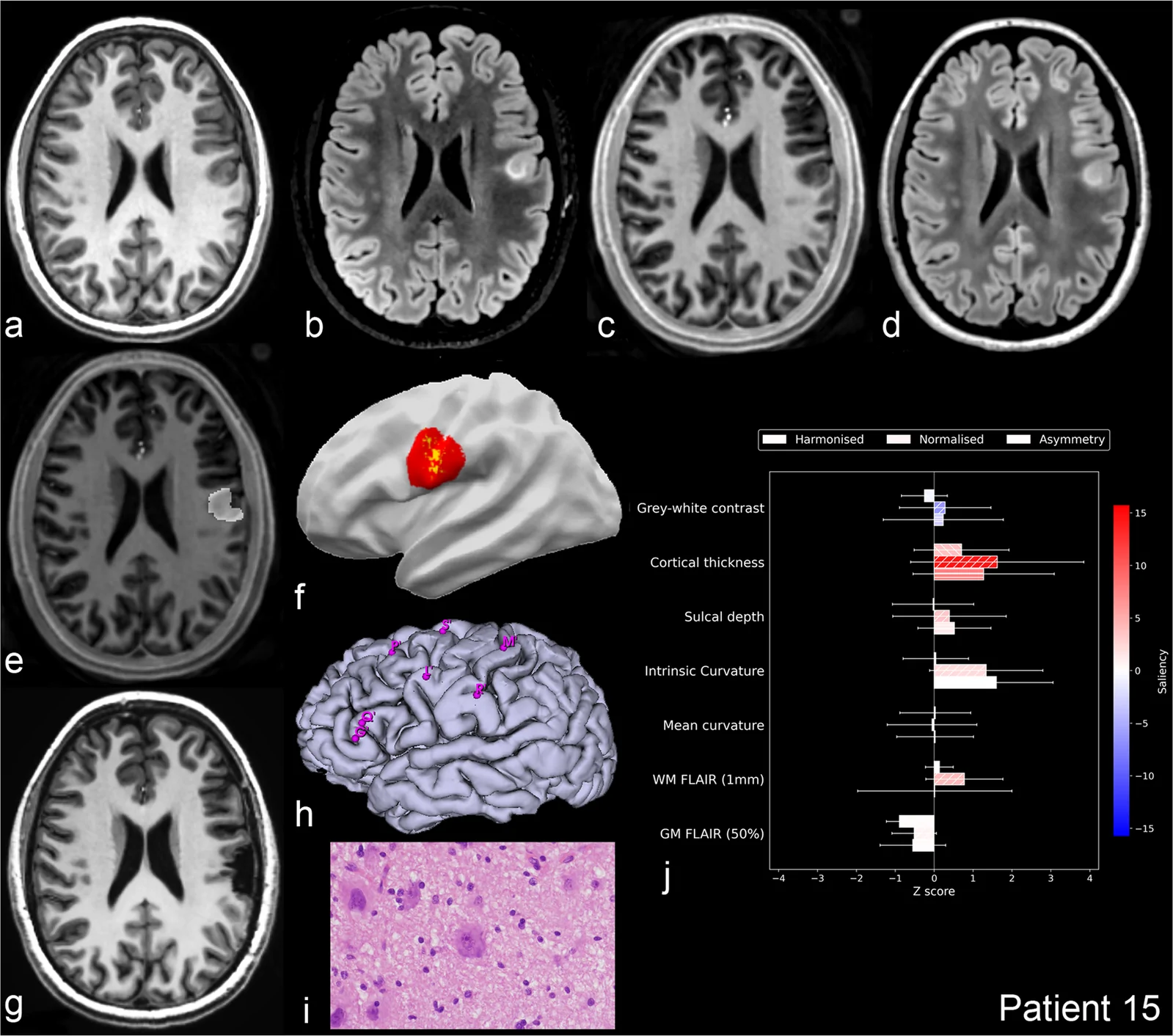
Preoperative imaging, intracranial stereo-EEG, surgical outcome, and 7 T classifier results in a patient with FCD-IIb (Patient 15). Preoperative 3 T T1w (**a**) and FLAIR (**b**) MRI, and corresponding 7 T T1w (**c**) and FLAIR (**d**) MRI; 7 T T1w MRI (**e**) and 3D cortical surface model (**f**) with superimposed the lesion cluster detected by the 7 T classifier (white overlay in **e**; red-yellow overlay in **f**); postsurgical 3 T T1w MRI (**g**); 3D *pial* surface model of the left hemisphere with superimposed stereo-EEG electrode entry points (**h**); histopathology (**i**); 7 T classifier feature profile (**j**) of the lesion cluster. Axial 3 T (**a** and **b**) and 7 T (**c** and **d**) MRI demonstrate a dysplastic area with a transmantle sign, suggestive of FCD–II. The 7 T classifier accurately identified the lesion (**e** and **f**) and quantified its morphometric profile (**j**) characterized by increased cortical thickness, sulcal depth, intrinsic curvature, and white-matter FLAIR signal. These features helped classifying the lesion as FCD, before it was histologically confirmed to be FCD-IIb (**i**), as shown by a centrally located dysmorphic neuron with abundant coarse Nissl substance, flanked by balloon cells with eccentric nuclei and eosinophilic cytoplasm (hematoxylin-eosin stain; original magnification 20x)

**Fig. 4:**
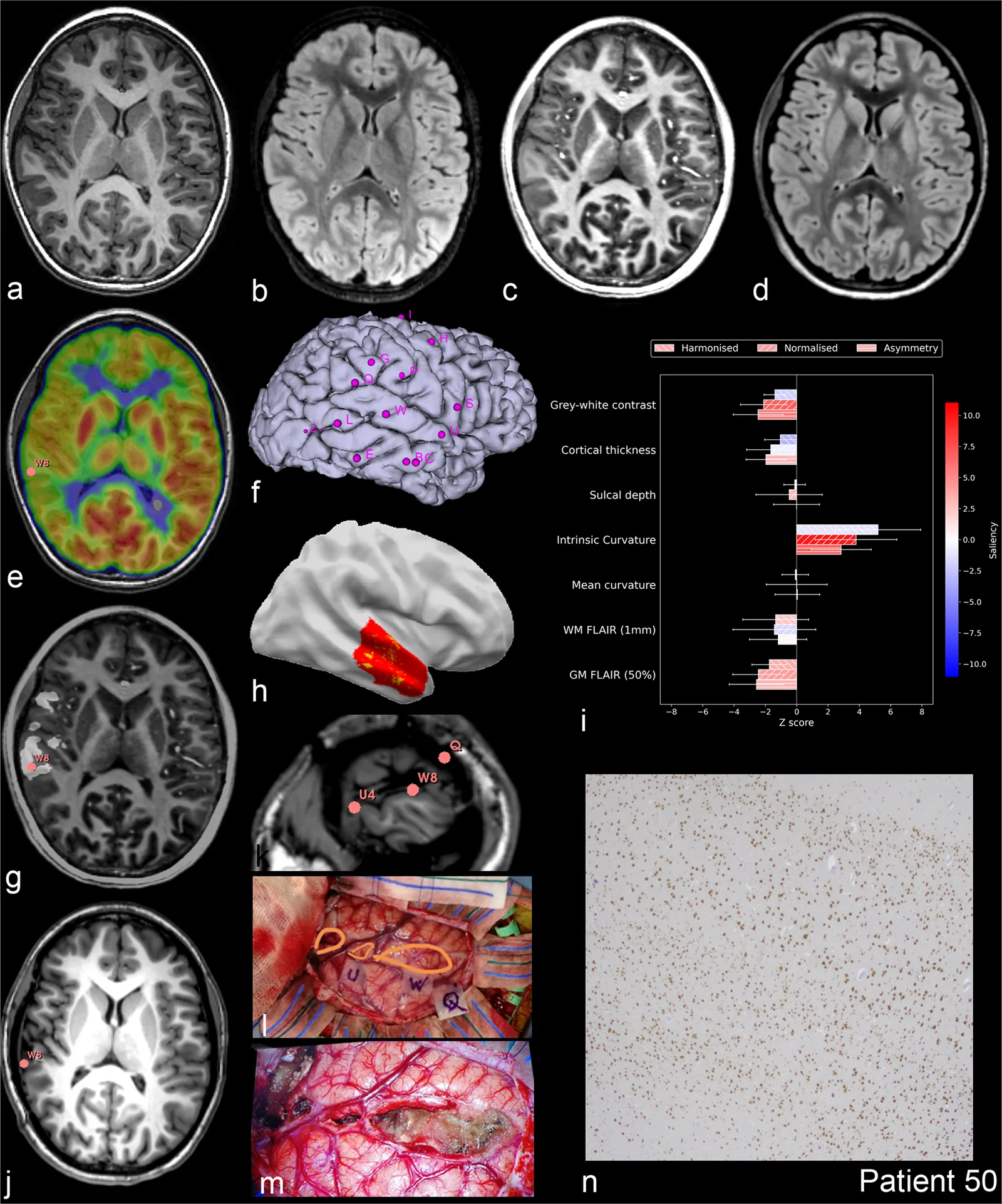
Preoperative imaging, FDG-PET, intracranial stereo-EEG, surgical outcome, and 7 T classifier results in a patient with FCD-Ib (Patient 50). Preoperative 3 T T1w (**a**) and FLAIR (**b**) MRI, corresponding to 7 T T1w (**c**) and FLAIR (**d**) MRI; 3 T T1w MRI co-registered with the FDG-PET imaging and superimposed stereo-EEG contacts involved in the SOZ (**e**); 3D *pial* surface model of the right hemisphere with superimposed stereo-EEG electrode entry points (**f**); 7 T T1w MRI (**g**) and 3D cortical surface model (**h**) with superimposed the lesion cluster detected by the 7 T classifier (white overlay in **g**; red-yellow overlay in **h**); 7 T classifier feature profile (**i**) of the lesion cluster; axial (**j**) and sagittal (**k**) post-surgical 3 T T1w MRI with superimposed stereo-EEG contacts involved in the SOZ; intraoperative photographs of the temporal lobe corticectomy (**l** and **m**); histopathological findings (**n**). In the preoperative axial 3 T (**a** and **b**) and 7 T (**c** and **d**) MRI there was not a clear detectable lesion, although FDG-PET imaging (**e**) revealed hypometabolism in the right temporal region. The 7 T classifier identified a lesion cluster (**g** and **h**) and quantified its morphometric profile (**i**), characterized by altered grey-white matter contrast, cortical thickness, sulcal depth, intrinsic curvature, and white matter and grey matter FLAIR signal. Following stereo-EEG evaluation (**f**), the patient underwent surgical resection (**j**-**m**). The morphometric features supported the diagnosis of FCD, which was confirmed as FCD-Ib by histopathology (**n**), with mild disorganization of cortical layers and subtle abnormalities of neuronal distribution (NeuN immunostain; original magnification 20x)

**Fig. 5:**
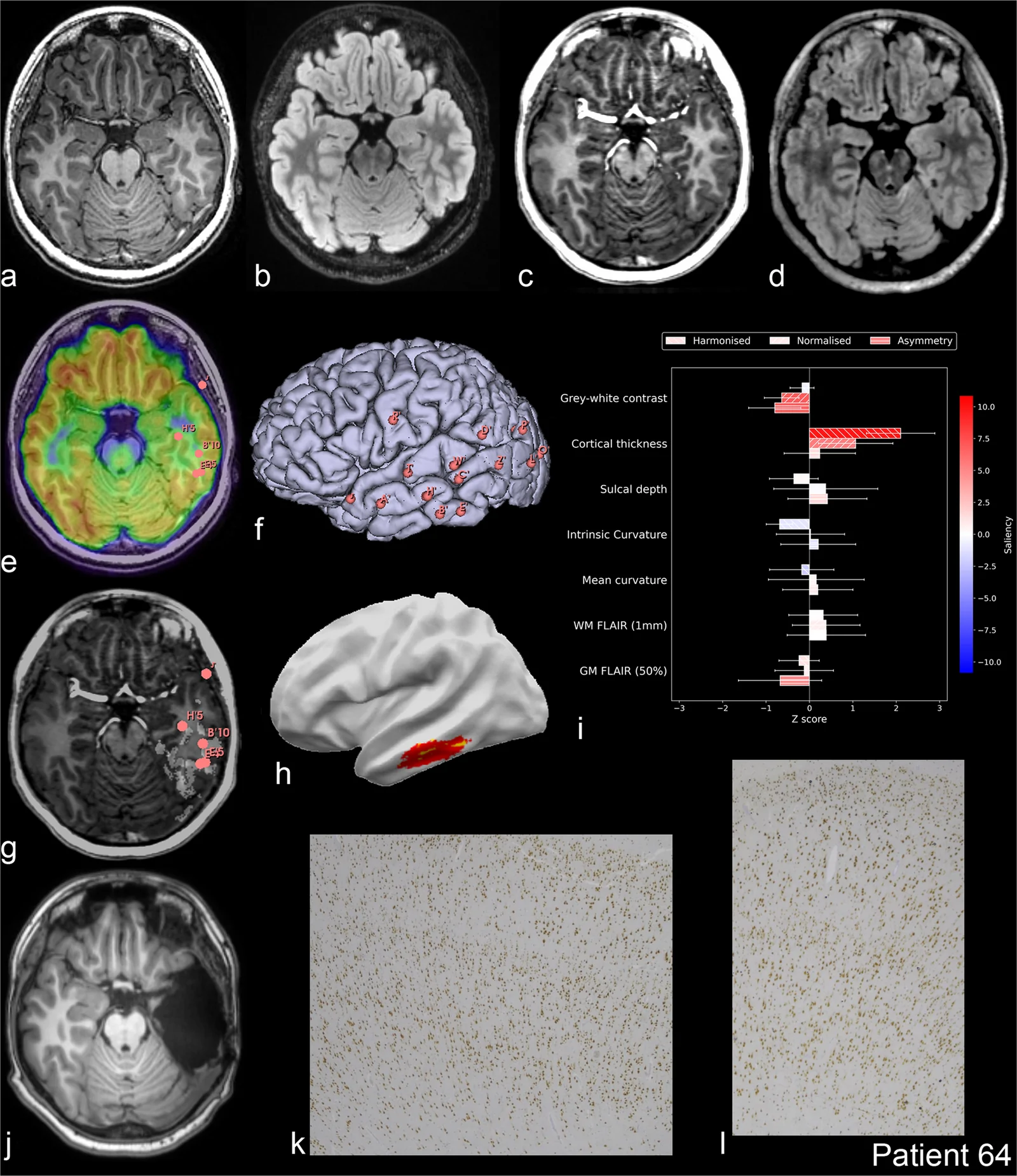
Preoperative imaging, FDG-PET, intracranial stereo-EEG, surgical outcome, and classifier results in a patient with no definite FCD on histopathology (Patient 64). 3 T T1w (**a**) and FLAIR (**b**) MRI, corresponding to 7 T T1w (**c**) and FLAIR (**d**) MRI, and 3 T T1w MRI with co-registered FDG-PET imaging and superimposed stereo-EEG contacts involved in the SOZ (**e**); 3D *pial* surface model of the left hemisphere with superimposed stereo-EEG electrode entry points (**f**); 7 T T1w MRI (**g**) and 3D cortical surface model (**h**) with superimposed the lesion cluster detected by the 7 T classifier (white overlay in **g**; red-yellow overlay in **h**); 7 T classifier feature profile (**i**) of the lesion cluster; axial postsurgical 3 T T1w MRI (**j**); histopathological findings (**k** and **l**). Preoperative axial 3 T (**a** and **b**) and 7 T (**c** and **d**) MRI were unrevealing, while FDG-PET imaging (**e**) demonstrated hypometabolism in the left temporal lobe. The 7 T classifier identified a lesion cluster in the left temporal lobe (**g** and **h**), and quantified its morphometric profile (**i**), characterized by increased cortical thickness, blurring of the grey-white matter junction and altered grey and white matter signals on FLAIR. Following stereo-EEG (**f**), the patient underwent left temporal lobectomy (**j**). The morphometric features are suggestive of an FCD-like lesion, which was confirmed by histopathology (left temporal pole, **k**; posterior temporal lobe, **l**) showing mild-to-moderate abnormal neuronal distribution and laminar disorganization, consistent with no definite FCD at histopathology (NeuN immunostain; original magnification 20x)

In 25/37 (67.6%) patients with 3 T MRI-positive findings (Table 1), 7 T imaging classification was concordant with 3 T visual assessment (Figs. 3 and 4). In 15/33 (45.4%) 3 T MRI-negative patients, the 7 T lesion classifier identified a cluster in a region concordant with the clinical and neurophysiological localization (Fig. 5).

**Table 1:** 7 T classifier performance in 3 T MRI-positive and MRI-negative patients

Patients	3 T MRI-positive	3 T MRI-negative	Total
	37/70 (52.9%)	33/70 (47.1%)	70/70 (100%)
Concordance between 7 T lesion cluster and visual MRI, electroclinical localization, or both	25/37 (67.6%)	15/33 (45.4%)	40/70 (57.1%)

In 7/10 (70.0%) patients who underwent intracranial stereo-EEG investigations, lesion clusters detected at 7 T showed spatial concordance with the stereo-EEG-defined SOZ (Figs. 4 and 5).

Seventeen of 70 (24.3%) patients underwent epilepsy surgery and the 7 T classifier detected histopathologically-confirmed lesions in 11/17 (64.7%). Histopathology revealed FCD-I in two patients, FCD-II in eight, FCD-III in two, and no definite FCD on histopathology in five. The 7 T lesion classifier correctly detected a lesion in 1/2 (50.0%) patients with FCD-I (e.g., Patient 50, Fig. 4), 5/8 (62.5%) patients with FCD-II (e.g., Patient 15, Fig. 3), and no patients with FCD-III. Among patients with no definite FCD on histopathology, the 7 T classifier correctly identified a lesion in 5/5 (100.0%) (e.g., Patient 64, Fig. 5).

In most 3 T MRI-negative patients, spatial concordance was based solely on clinical semiology and EEG findings and, in a limited number of individuals, on stereo-EEG examinations and post-surgical histopathology. Although validation based on stereo-EEG and histopathology would provide a more robust spatial reference, restricting the analysis exclusively on these patients would substantially reduce the size of the evaluable cohort, as only 3/30 patients with negative 3 T MRI underwent stereo-EEG and only 2/3 subsequent epilepsy surgery, thereby limiting the interpretability of the results. Nevertheless, the concordance with stereo-EEG and histopathological findings supports the potential clinical relevance of the 7 T surface-based GNN pipeline as an adjunctive tool for investigating the SOZ in surgical candidates with inconclusive MRI, and may help guide stereo-EEG planning in selected individuals.

### Comparison of lesion classification between 7 T and 3 T MRI

The 7 T classifier identified a lesion cluster concordant with either 3 T MRI, clinical-neurophysiological features, or both, in 40/70 (57.1%, 25 MRI-positive and 15 MRI-negative) patients (Table 2). Lesion detection outcome is illustrated in the flowchart reported in Supplementary Fig. 2.

**Table 2:** Metrics for the comparison of lesion detection performance between 7 T and 3 T MRI

Lesion detection outcome		No. of patients/subtotal	%
Patients detected by 7 T classifier		40/70	57.1%
	Exclusively detected by 7 T classifier	15/40	37.5%
	Improved conspicuity by 7 T classifier than 3 T classifier	11/40	27.5%
	Similar performance between 7 T and 3 T classifiers	14/40	35.0%
Patients with inconclusive 7 T classification		30/70	42.9%
	Not detected also by 3 T classifier	25/30	83.3%
	Exclusively detected by 3 T classifier	5/30	16.7%

**Fig. 6:**
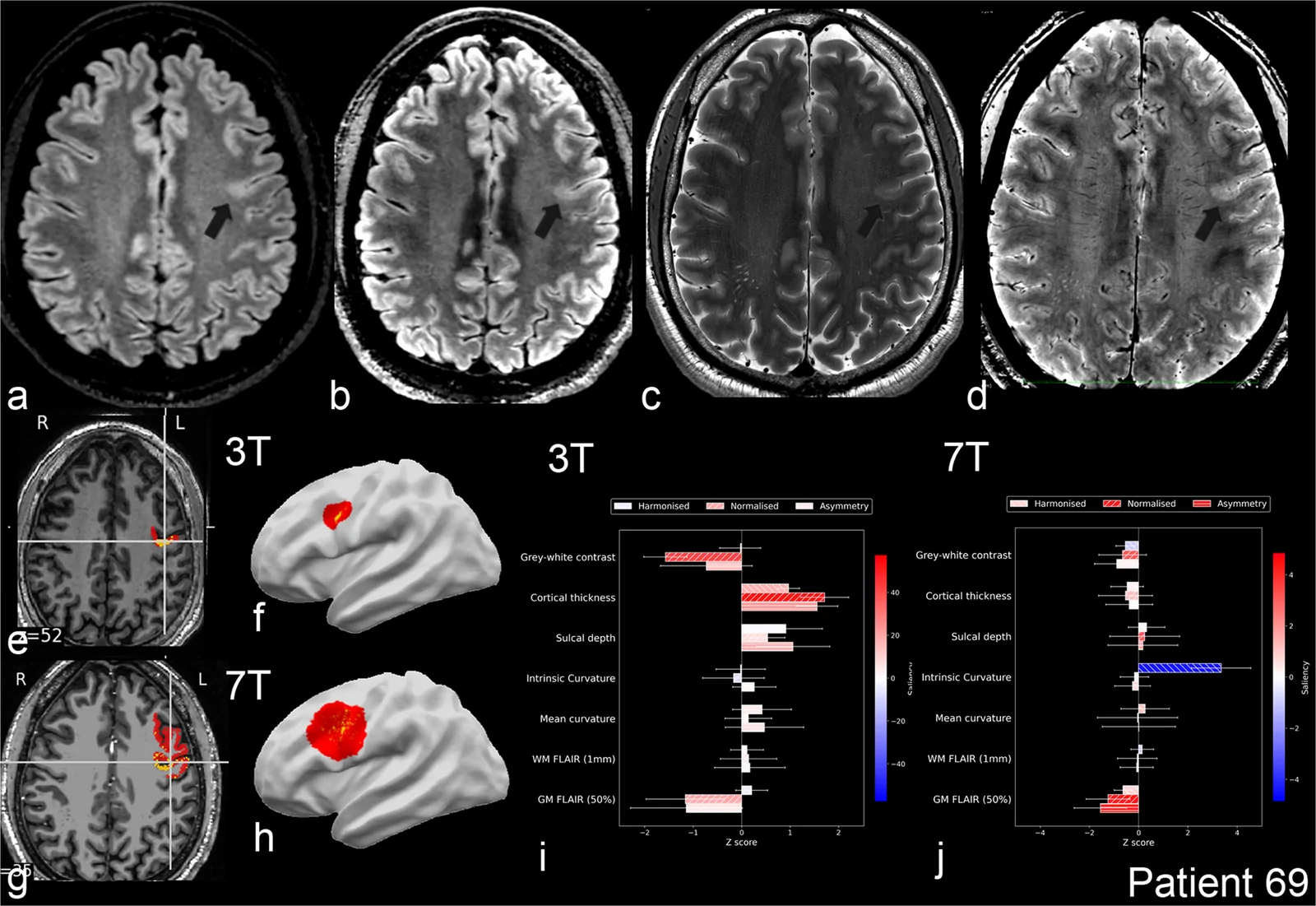
Comparison of 3 T and 7 T classifier results in an 3 T MRI-negative patient (Patient 69). Axial 3 T (**a**) and 7 T (**b**) FLAIR MRI, 7 T T2w (**c**) and Susceptibility-Weighted Angiography (SWAN) (**d**) MRI; axial 3 T T1w MRI (**e**) and the corresponding cortical surface 3D model (**f**) with the lesion cluster detected by the 3 T classifier (red-yellow overlay); axial 7 T T1w MRI (**g**) and the corresponding cortical surface 3D model (**h**) with the lesion cluster detected by the 7 T classifier (red-yellow overlay); 3 T (**i**) and 7 T (**j**) feature profiles of the clusters generated by the 3T- and 7T-based classifiers. Both the 3 T and 7 T classifiers identified a lesion cluster in the left frontal cortex (**e**-**h**), characterized by altered grey-white matter contrast and grey-matter FLAIR signal (**i** and **j**). The 3 T classifier revealed also a significant cortical thickening (**i**) not detected by the 7 T classifier (**j**)

In 15/40 (37.5%) patients lesion clusters could be detected by 7 T classifier but not at 3T. In 14/40 (35.0%) patients, lesion classification results were comparable between 7 T and 3T. In the remaining 11/40 (27.5%) patients, whose structural lesions were detected at 3 T, the 7 T classifier demonstrated improved lesion conspicuity, with clearer cortical involvement and/or larger extent of abnormal cortex compared with 3T-derived features. In some individuals, lesion clusters showed similar spatial extent at both field strengths (see Patient 69 shown in Fig. 6), although the features driving cluster significance differed substantially. Lesion clusters detected at 3 T were more frequently characterized by cortical thickening, whereas 7 T lesion clusters more commonly featured altered grey-white contrast or focal FLAIR abnormalities, likely reflecting a higher spatial resolution of 7 T imaging [41].

Among the 33/70 3 T MRI-negative patients, the 7 T classifier identified lesion clusters concordant with clinical and EEG localization in 15 (45.4%), including eight (53.3%) in whom no lesion was detected by the 3 T classifier.

In the 30/70 (37.0%) patients in whom the 7 T classifier did not identify a lesion cluster, 25/30 (83.3%) were negative also at 3T.

In 1/2 patients with histologically confirmed FCD-I, the 7 T cluster classification correctly detected the lesion region, whereas the 3 T classifier did not (Supplementary Table l). In patients with histopathologically confirmed FCD-II (8 patients), the 7 T lesion classifier demonstrated improved performance in lesion cluster detection compared with the 3 T classifier. In 1/2 patients with histologically confirmed FCD-III, the 3 T cluster classification correctly detected the lesion region, whereas the 7 T classifier did not. A higher detection yield was also observed in 2/5 patients with no definite FCD on histopathological examination.

**Fig. 7:**
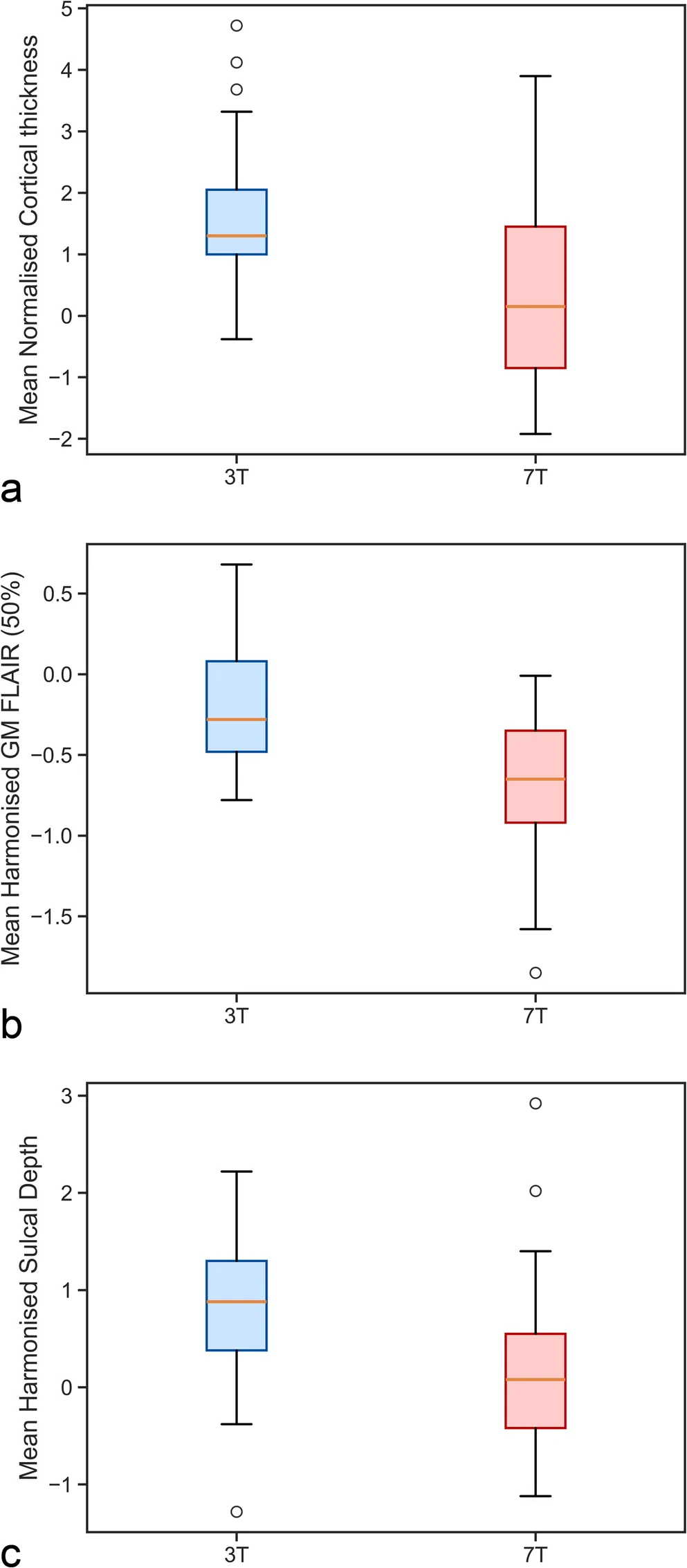
Comparison of 3 T and 7 T classifier results in the distribution of morphometric features. We observed a statistically significant reduction in the 7 T lesion clusters for the normalised cortical thickness (**a**), harmonised grey-matter (GM) FLAIR signal (**b**), and harmonised sulcal depth (**c**)

Overall, quantitative comparison of lesion cluster features between 7 T and 3 T revealed a trend toward reduced cortical thickness in clusters identified at 7 T relative to 3 T (Fig. 7). Statistically significant reductions in the 7 T lesion clusters were observed for normalized cortical thickness (t = 3.056, *p* = 0.036), harmonized gray-matter FLAIR signal (t = 3.684, *p* = 0.011), and harmonized sulcal depth (t = 3.223, *p* = 0.024).

## Discussion

Our study demonstrates that ultra-high-field 7 T MRI, combined with surface-based post-processing, provides a meaningful improvement over conventional 3 T MRI in the better characterization or de novo detection of subtle FCD or FCD-like lesions in a pediatric-predominant cohort with focal epilepsy.

Consistent with prior reports [14–18], visual assessment at 7 T MRI increases the diagnostic yield for focal structural brain abnormalities compared with 3 T MRI, particularly for subtle cortical abnormalities that may escape conventional imaging. While the magnitude of improvement varies across studies, our findings reinforce the clinical value of 7 T MRI as a complementary tool in focal epilepsy [14–18].

Beyond visual inspection, post-processing at 7 T MRI further enhanced lesion detection with respect to 3 T post-processing. In 3 T MRI-positive patients, even when comparing post-processing data, 7 T provided a more detailed delineation of lesion extent. Integrating 7 T imaging with surface-based post-processing enabled a quantitative characterization of lesion morphometry properties, complementing expert neuroimaging assessment. Rather than replacing visual analysis, automated classification acted as an adjunct, providing quantitative objective metrics of cortical thickness, grey-white matter contrast, and signal abnormalities to an extent that may support clinical decision-making and improve confidence in lesion boundaries [42].

Surface-based GNN classifier identified lesion clusters in 45.4% of 3 T MRI-negative patients, with strong spatial concordance with clinical semiology and EEG data. Although concordance in 3 T MRI-negative patients was based primarily on clinical and surface EEG findings, only a minority of these patients underwent intracranial stereo-EEG investigations and epilepsy surgery, limiting the feasibility of restricting the analysis exclusively to histopathologically validated individuals. However, in 3 T MRI-negative patients who underwent epilepsy surgery, areas highlighted by the 7 T classifier corresponded to histopathologically confirmed FCD. These findings are relevant in light of the challenges when considering MRI-negative patients, who even after extensive and invasive investigations, which may include stereo-EEG, still experience lower rates of seizure freedom [4, 5]. Our results suggest that advanced non-invasive imaging and post-processing may help reduce this diagnostic gap by improving localization of putative epileptogenic cortex in patients with suspected but imaging occult structural lesions.

Our results are in line with prior voxel-based studies applying the Morphometric Analysis Program (MAP) [21, 43] to 7 T MRI, which reported a comparable increase in diagnostic yield in MRI-negative patients. However, the surface-based framework we adopted differs conceptually and methodologically from MAP. By reconstructing high-resolution cortical surfaces and sampling features across multiple cortical depths, morphometric and intensity-based abnormalities captured by our approach more closely reflect cortical architecture. GNN [30] further enables the modeling of non-linear and spatially contextual relationships between features [44], potentially allowing for more patient-specific representations of the dysplastic cortex. This approach contrasts with voxel-wise, group-level statistical framework used by MAP, in which each individual’s features are compared with those of healthy controls to identify statistically deviant regions [45]. While MAP has proven effective and widely applicable, it remains inherently dependent on population-level thresholds and may be less sensitive to subtle, patient-specific patterns of cortical dysplasia. Although both MAP [21] and the GNN-based classifier can operate on high-resolution 7 T T1w data, our approach additionally integrates 3D FLAIR imaging, enabling the detection of cortical and subcortical signal abnormalities frequently observed in FCD, particularly type II.

Differences in diagnostic yield between studies likely reflect a combination of methodological distinctions, including feature selection, spatial modeling, and classification strategy, as well as cohort characteristics. The data-driven training of the GNN allows the model to learn complex feature interactions directly from labeled examples, rather than relying on predefined statistical contrasts. This strategy may help identifying more granular and patient-specific disease signatures, potentially enhancing applicability in individualized evaluation of children with focal epilepsy and suspected dysplastic lesions, especially in MRI-negative patients where conventional imaging and voxel-based methods may fail to capture subtle abnormalities [46].

We adopted the GNN algorithm developed and extensively validated within the MELD project, a large multicenter initiative involving 22 epilepsy centers worldwide and a cohort of 1,015 participants (618 patients) scanned at 1.5T and 3 T MRI [29, 30]. In the full MELD cohort, the classifier achieved a sensitivity of 81.6% in histopathologically confirmed patients and 63.7% in MRI–negative patients with FCD. Several other automated MRI-based FCD detectors have been proposed [25], including those applied in two multicenter studies based on voxel-wise MRI analysis [26, 47]. The MELD classifier relies on FreeSurfer [38], a robust and widely adopted pipeline for cortical surface reconstruction, openly available as a Python package on GitHub, and validated on a large, heterogeneous real-world dataset, including 256 pediatric patients and 44 FCD-I. In our predominantly pediatric cohort, 7 T implementation of the MELD classifier successfully detected 6/7 histologically confirmed FCD-I or no definite FCD lesions [31]. In addition to detection performance, the MELD framework provides an intuitive and clinically meaningful visualization of results: for each lesion cluster, bar plots summarize the morphometric features contributing most to its saliency. This level of interpretability enables direct comparison with conventional visual MRI assessment and facilitates integration of computational detection tools into routine neuroimaging practice [48].

The 7 T surface-based GNN pipeline showed a limited burden of false-positive detections in our cohort. In most concordant individuals, the algorithm identified a single cluster, whereas detection of multiple clusters within the same patient was uncommon. This observation is relevant considering that a high false-positive rate represented a known limitation of the original MELD surface-based framework [29]. The improved specificity observed in our study may also reflect the advantages of the GNN architecture introduced in the updated MELD pipeline [30, 49], which was designed to better integrate relationships between neighboring cortical regions, thereby reducing unlikely lesion predictions. Overall, these findings further support the potential clinical utility of the 7 T surface-based GNN method in the presurgical evaluation of epilepsy patients.

Direct comparison of classifier performance at 7 T and 3 T demonstrated a consistent advantage of ultra-high field imaging. The 7T-based classifier detected 37.5% more lesion clusters than its 3T-based counterpart. In most patients where 7 T was unrevealing 3 T was also so, with five exceptions. In the subgroup of patients in whom both classifiers identified a lesion cluster, 27.5% had 7 T classification that provided greater visibility of the lesions and sharper delineation of their boundaries. These improvements likely reflect the higher spatial resolution and enhanced anatomical detail of 7 T MRI, which mitigates voxel-size-related partial-volume effects [18]. Lesion clusters detected by both approaches exhibited different feature profiles contributing to classifier saliency. While the 3T-based model more frequently emphasized cortical thickening, the 7 T classifier more often highlighted reduced grey-white matter contrast and focal signal abnormalities. This difference is consistent with previous demonstrations that cortical thickness is more accurately measured at ultra-high-field [41], enabling a more refined characterization of the dysplastic cortex [20]. From an imaging perspective, FCD-I typically presents with cortical thinning, grey-white matter blurring, and occasionally lobar hypoplasia, whereas FCD-II more commonly features cortical thickening, signal changes and abnormal gyrification [23]. In our cohort, this distinction was reflected in the classifier behavior: while cortical thickening contributed prominently to lesion detection at 3 T, the 7 T classifier more frequently relied on grey-white matter blurring and FLAIR abnormalities. Improved spatial resolution at 7 T may reduce artificial augmentation of cortical thickness measurements [41], shifting feature weighting toward contrast- and signal-based markers that more specifically capture lesion architecture. Consequently, surface-based classification at 7 T may better discriminate between FCD subtypes, an effect that was particularly advantageous when investigating MRI-negative patients, where gains in lesion conspicuity were most pronounced.

Improved a priori lesion detection may ultimately facilitate clinical management including, when appropriate, surgical planning by reducing reliance on invasive diagnostic procedures.

Several limitations should be acknowledged. First, the MELD classifier was originally trained through large 1.5T and 3 T datasets. Although we applied harmonization procedures, dedicated training on 7 T data is not yet feasible, largely due to the limited availability of pediatric patients with clearly delineated lesions; in our cohort, only 49/87 individuals had a visible lesion at 3 T MRI. An additional potential limitation is related to the increased susceptibility of ultra-high-field MRI to signal non-uniformity and spatial distortions, which are especially pronounced in the temporal areas and may affect the reliability of measurements in these regions, increase the patients’ exclusion rate, and limit the clinical translation of post-processing MRI prototypes. While these effects were partially mitigated through recent hardware and software upgrades and the inclusion of dedicated correction steps in our processing pipeline, residual variability may persist. The GNN pipeline concordance rate was observed in patients who were able to tolerate high-quality, motion-free scan and whose data successfully completed the surface-based reconstruction pipeline. Finally, we acquired 3 T examinations at two different centers, and collected 7 T data across subsequent scanner upgrades, a technical variability which we compensated by applying established harmonization strategies to limit its impact. We also acknowledge that applying a GNN trained on 3 T data to 7 T scans involves a change of domain, which could affect the classifier performance despite harmonization. However, post-harmonization analysis of the feature distributions supported the effectiveness of our approach, revealing a substantial overlap of the main distribution peaks while preserving specific field-strength-related features.

Looking forward, several developments may further enhance the performance and clinical applicability of surface-based classifiers at ultra-high field. Fine-tuning of pretrained classifiers directly on 7 T data, integrating additional MRI contrasts – such as SWAN or GRE, which have demonstrated high sensitivity for FCD at 7 T [15] – and expanding multicenter collaborations to build larger, exclusively 7 T training dataset are likely to improve model robustness and generalizability. Multi-contrast post-processing frameworks may further increase detection yield by leveraging complementary tissue signatures across sequences [28, 50, 51]. Ongoing technical advances, including parallel transmit systems and dedicated radiofrequency coils, may further mitigate B1 inhomogeneity and improve image quality in challenging brain regions.

## Conclusion

By combining ultra-high-field MRI with surface-based reconstruction and an advanced, extensively validated GNN classifier, we demonstrated a diagnostic benefit of 7 T MRI with post-processing in identifying focal structural brain abnormalities in patients with focal epilepsy and suspected subtle cortical lesions. Surface-based analysis at 7T mitigates voxel-related partial-volume effects and enhances lesion detection and conspicuity, particularly in MRI-negative patients. These findings support the broader clinical adoption of 7 T MRI with advanced post-processing as a valuable tool in the diagnostics of subtle cortical abnormalities.

## Supplementary Information

Below is the link to the electronic supplementary material.

Supplementary Material 1 (DOCX 10.6 MB)

## Data Availability

The data are available from the authors upon reasonable request.
