# Supplementary material for: Enhanced detection of subtle cortical abnormalities in focal epilepsy using 7 T MRI surface-based models and graph neural networks
Source: Neuroradiology. 2026 Jul 6;68(8):2241–56. doi: 10.1007/s00234-026-04103-8 (PMC13577987; doi:10.1007/s00234-026-04103-8)
Supplement: Supplementary file 1 [file 234_2026_4103_MOESM1_ESM.docx]

**Supplementary Material**

| **Pt #** | **Sex** | **Age (y)** | **EEG interictal** | **EEG ictal** | **PET** | **SEEG** | **Surgery** | **Histopathology** | **Age at 3T MRI** | **Age at 7T MRI** | **3T visual review according to Desikan et al. [1]** | **7T visual review** | **7T classifier vs visual concordance / QC (failure mode)** | **7T classifier vs EEG abnormalities concordance** | **7T vs 3T classifier comparison** | **No. of clusters** |
| --- | --- | --- | --- | --- | --- | --- | --- | --- | --- | --- | --- | --- | --- | --- | --- | --- |
| 1 | F | 28 | SPK: L FC, R F, R TO | Na | Nd | Nd | Nd | Ns | 17 | 18 | L lateral occipital; L pericalcarine | Same as 3T | Negative | Nd | - | 0 |
| 2 | M | 17 | IFS: L P  SPK: L P | Na | Nd | Nd | Nd | Ns | 16 | 16 | L superior frontal | Same as 3T | QC (segmentation error) | Nd | Na | Na |
| 3 | F | 34 | SPK: L hemispheric | Na | Nd | Nd | Nd | Ns | 20 | 27 | L hemispheric | Same as 3T | Positive | Nd | Similar | 1 |
| 4 | M | 9 | SWC: L PO, Bi CT | Na | Nd | Nd | Nd | Ns | 7 | 8 | L superior frontal; L temporal pole | Same as 3T | Negative | Nd | - | 0 |
| 5 | M | 14 | SPK: Bi Fr  IFS: R Fr | Na | Nd | Nd | Needle Biopsy | FCD IIb | 9 | 11 | L temporal pole | Same as 3T | QC (T1w movement artifacts) | Nd | Na | Na |
| 6 | M | 22 | SPK: L FC, R Fr | NA | Nd | Nd | Nd | Ns | 12 | 19 | L precentral | Same as 3T | QC (T1w movement artifacts) | Nd | Na | Na |
| 7 | F | 14 | SPK: L FC Ver | L F | Nd | Nd | Yes | FCD IIb | 10 | 10 | L superior frontal; L caudal anterior cingulate | Same as 3T | Positive | Nd | ++ | 1 |
| 8 | M | 21 | SWC: L CT | Na | Nd | Nd | Nd | Ns | 10 | 10 | L precentral | Same as 3T | QC (segmentation error) | Nd | Na | Na |
| 9 | M | 13 | SPK: L FCT | Na | Nd | Nd | Nd | Ns | 16 | 11 | L temporal pole; L amygdala; L entorhinal; L parahippocampal | Same as 3T | Positive | Nd | ++ | 1 |
| 10 | F | 14 | SPK: Bil FC (L>R), Ant Ver | Na | Nd | Nd | Nd | Ns | 10 | 12 | L inferotemporal | Same as 3T | QC (T1w movement artifacts) | Nd | Na | Na |
| 11 | F | 23 | SWC: L FCT | L FT | Nd | Nd | Nd | Ns | 13 | 13 | L temporal pole; L amygdala; L hippocampus | Same as 3T | Positive | Nd | + | 1 |
| 12 | M | 30 | SPK: L FCT | L FC | Nd | Nd | Nd | Ns | 14 | 19 | L hippocampus; L amygdala; L temporal pole | Same as 3T | QC (T1w MRI movement artifacts) | Nd | Na | Na |
| 13 | M | 33 | SPK: R T | Na | Yes | Yes | Yes | FCD IIIa | 22 | 22 | R hippocampus; R temporal pole | Same as 3T | Negative | Nd | - | 0 |
| 14 | F | 42 | SW: Bil Fr | Na | Nd | Nd | Nd | Ns | 31 | 31 | L rostral middle frontal; L medial orbitofrontal | Same as 3T | QC (segmentation error) | Nd | Na | Na |
| 15 | F | 26 | SPK: L FC | Na | Nd | Yes | Yes | FCD IIb | 17 | 17 | L precentral | Same as 3T | Positive | Nd | Similar | 1 |
| 16 | F | 21 | SPK: R PO | Na | Nd | Nd | Nd | Ns | 10 | 11 | R pericalcarine | Same as 3T | Negative | Nd | Not detected | 1* |
| 17 | F | 23 | SPK: Bil Fr (R>L) | Na | Nd | Yes | Yes | FCD IIb | 12 | 12 | R rostral middle frontal; R pars triangularis | Same as 3T | Negative | Nd | Not detected | 0 |
| 18 | M | 17 | SW and SPK: L T | Na | Nd | Nd | Nd | Ns | 12 | 16 | L hippocampus; L amygdala | Same as 3T | Negative | Nd | Not detected | 1* |
| 19 | F | 18 | SWC: R FT | Na | Nd | Nd | Nd | Ns | 13 | 15 | R supramarginal; R inferior parietal | Same as 3T | Positive | Nd | + | 1 |
| 20 | M | 34 | SPK: L FC | Na | Nd | Nd | Nd | Ns | 9 | 9 | L rostral middle frontal | Same as 3T | Positive | Nd | Similar | 1 |
| 21 | M | 21 | SWC: R Fr | R Fr | Nd | Nd | Nd | Ns | 22 | 24 | R rostral middle frontal | Same as 3T | Positive | Nd | + | 1 |
| 22 | M | 19 | SPK: L FCT | Na | Nd | Nd | Yes | ND FCD | 12 | 13 | L amygdala; L hippocampus; L temporal pole | Same as 3T | Positive | Nd | Similar | 1 |
| 23 | M | 19 | SPK: R TP, R PO | R TO | Nd | Nd | Yes | FCD IIa | 9 | 9 | R precuneus; R superior parietal | Same as 3T | QC (FLAIR MRI movement artifacts) | Nd | Na | Na |
| 24 | M | 18 | SWC: R FCP | R FrCP | Yes | Yes | Yes | ND FCD | 11 | 11 | R postcentral | Same as 3T | Positive | Nd | Similar | 1 |
| 25 | M | 21 | SPK: R FT | Na | Nd | Nd | Nd | Ns | 7 | 8 | R hippocampus; R amygdala; R temporal pole | Same as 3T | Positive | Nd | ++ | 1 |
| 26 | M | 27 | None | Na | Nd | Nd | Nd | Ns | 7 | 11 | L hippocampus; L temporal pole; L fusiform | Same as 3T | Negative | Nd | Not detected | 0 |
| 27 | M | 20 | SPK: FCT | Na | Nd | Nd | Nd | Ns | 15 | 25 | R precentral; R superior frontal | Same as 3T | Positive | Nd | ++ | 1 |
| 28 | M | 19 | SW and SPK: L Ant FCT | Na | Nd | Yes | Yes | FCD IIb | 12 | 12 | L pars triangularis; L pars opercularis | Same as 3T | Positive | Nd | Similar | 1 |
| 29 | M | 17 | SPK: Bil FCT (R>>L) | Na | Nd | Nd | Nd | Ns | 12 | 13 | L supramarginal; L postcentral | Same as 3T | Positive | Nd | Similar | 1 |
| 30 | F | 20 | None | Na | Nd | Nd | Nd | Ns | 7 | 8 | L inferior temporal; L middle temporal | Same as 3T | QC (T1w MRI movement artifacts) | Nd | Na | Na |
| 31 | F | 18 | SPK: Bil FC (L>R), Ver | R C, Ver | Nd | Nd | Nd | Ns | 17 | 17 | R caudal middle frontal | Same as 3T | Positive | Nd | ++ | 1 |
| 32 | M | 18 | SPK: L Fr, L T | Na | Nd | Nd | Nd | Ns | 15 | 12 | L caudal middle frontal | Same as 3T | Positive | Nd | Similar | 1 |
| 33 | M | 16 | SWC and SW: R Fr, Ant Ver | Na | Nd | Yes | Yes | FCD IIb | 17 | 16 | R superior frontal | Same as 3T | Positive | Nd | Similar | 1 |
| 34 | M | 19 | SW: R Ant FrT, Ver | Na | Nd | Nd | Yes | FCD IIb | 9 | 9 | R rostral middle frontal | Same as 3T | Positive | Nd | + | 1 |
| 35 | F | 23 | SPK: R O | Na | Nd | Nd | Nd | Ns | 6 | 9 | R lateral occipital; R pericalcarine | Same as 3T | Negative | Nd | Not detected | 0 |
| 36 | F | 18 | SPK: Bil TPO, L T, Bil FT | Na | Nd | Nd | Nd | Ns | 16 | 20 | L temporal pole; L middle temporal | Same as 3T | Negative | Nd | Not detected | 0 |
| 37 | F | 20 | SWC: L T | L T | Nd | Nd | Yes | FCD Ib | 9 | 10 | L temporal pole | Same as 3T | Negative | Nd | Not detected | 0 |
| 38 | F | 25 | SWC: R Fr, R OF | Na | Yes | Yes | Yes | FCD IIa | 8 | 10 | R lateral orbitofrontal; R pars orbitalis | Same as 3T | Negative | Nd | Not detected | 0 |
| 39 | F | 16 | SPK: L FT | Na | Nd | Nd | Yes | ND FCD | 16 | 16 | L entorhinal; L temporal pole; L inferior temporal; L middle temporal; L amygdala; L hippocampus | Same as 3T | QC (T1w MRI movement artifacts) | Nd | Na | Na |
| 40 | M | 47 | SPK: L FT | Na | Yes | Nd | Nd | Ns | 13 | 13 | L amygdala; L hippocampus | Same as 3T | Negative | Nd | Not detected | 0 |
| 41 | F | 19 | SPK: R FCT | Na | Nd | Nd | Nd | Ns | 45 | 45 | L superior frontal | Same as 3T | Positive | Nd | + | 1 |
| 42 | F | 38 | SW: L TP | Na | Nd | Nd | Nd | Ns | 10 | 8 | L inferior parietal | Same as 3T | Positive | Nd | ++ | 1 |
| 43 | F | 11 | SPK: R FC | Na | Nd | Nd | Nd | Ns | 37 | 37 | R precentral; R postcentral | Same as 3T | QC (FLAIR MRI movement artifacts) | Nd | Na | Na |
| 44 | M | 13 | SPK: L FT | L FT | Nd | Nd | Nd | Ns | 4 | 8 | L insula | Same as 3T | Positive | Nd | + | 1 |
| 45 | F | 18 | SW: R C | Na | Nd | Nd | Nd | Ns | 8 | 10 | R caudal middle frontal; R precentral | Same as 3T | Positive | Nd | Similar | 1 |
| 46 | F | 22 | SPK: R T | Na | Nd | Nd | Nd | Ns | 8 | 8 | R middle temporal | Same as 3T | Positive | Nd | ++ | 1 |
| 47 | F | 18 | SPK: isolated Bil | Na | Nd | Nd | Nd | Ns | 13 | 15 | R insula | Same as 3T | Positive | Nd | ++ | 1 |
| 48 | F | 15 | SPK: R Fr, R O | Na | Nd | Nd | Nd | Ns | 10 | 11 | R superior frontal | Same as 3T | QC (FLAIR MRI movement artifacts) | Nd | Na | Na |
| 49 | M | 18 | SPK: L FCT | Na | Yes | Nd | Yes | ND FCD | 7 | 8 | L hippocampus; L temporal pole | Same as 3T | Positive | Nd | + | 1 |
| 50 | F | 18 | SPK: R FT | Na | Yes | Yes | Yes | FCD Ib | 9 | 10 | Normal | Normal | Nd | Positive | ++ | 1 |
| 51 | F | 32 | SPK: L T | Na | Nd | Nd | Nd | Ns | 17 | 19 | Normal | Normal | Nd | QC (no whole-brain acquisition) | Na | Na |
| 52 | M | 36 | SPK: Bil Fr (L>R) | L Fr | Nd | Nd | Nd | Ns | 19 | 24 | Normal | Normal | Nd | QC (no whole-brain acquisition) | Na | Na |
| 53 | F | 39 | SWC: L FT | L FT | Nd | Nd | Nd | Ns | 21 | 27 | Normal | Normal | Nd | QC (no whole-brain acquisition) | Na | Na |
| 54 | M | 51 | SPK and SWC: L FT | L FT | Nd | Nd | Nd | Ns | 42 | 40 | Normal | Normal | Nd | Positive | Similar | 1 |
| 55 | M | 24 | SPK: R FT | Na | Nd | Nd | Yes | FCD IIIa | 13 | 13 | Normal | Normal | Nd | Negative | Not detected | 0 |
| 56 | M | 33 | SWC: Bil FCT (R>L) | R FCT | Nd | Nd | Nd | Ns | 20 | 22 | Normal | Normal | Nd | Negative | Not detected | 1* |
| 57 | M | 24 | SPK: L PO | Na | Nd | Nd | Nd | Ns | 10 | 14 | Normal | Normal | Nd | Negative | Not detected | 0 |
| 58 | F | 23 | SPK: R FC | Na | Nd | Nd | Nd | Ns | 7 | 13 | Normal | Normal | Nd | Positive | ++ | 1 |
| 59 | F | 39 | SPK: R FT | Na | Nd | Nd | Nd | Ns | 28 | 35 | Normal | Normal | Nd | Positive | + | 1 |
| 60 | M | 18 | SPK: R FC | Na | Nd | Nd | Nd | Ns | 8 | 8 | Normal | Normal | Nd | Positive | Similar | 1 |
| 61 | M | 23 | SWC: L Fr | L Fr | Nd | Nd | Nd | Ns | 13 | 13 | Normal | Normal | Nd | Positive | + | 2* |
| 62 | F | 20 | SPK: L C | Na | Nd | Nd | Nd | Ns | 8 | 10 | Normal | Normal | Nd | Negative | Similar | 0 |
| 63 | M | 25 | SPK: L FT | Na | Nd | Nd | Nd | Ns | 17 | 17 | Normal | Normal | Nd | Positive | ++ | 1 |
| 64 | F | 18 | SPK: L TO | Na | Yes | Yes | Yes | ND FCD | 11 | 11 | Normal | Normal | Nd | Positive | ++ | 1 |
| 65 | M | 21 | SWC and SPK: L Fr, Ver | Bil Fr (L>R) | Nd | Nd | Nd | Ns | 7 | 14 | Normal | Normal | Nd | Positive | ++ | 1 |
| 66 | F | 19 | SPK: L Fr, Ant Ver | L Fr | Nd | Nd | Nd | Ns | 13 | 13 | Normal | Normal | Nd | Negative | - | 0 |
| 67 | M | 51 | SWC: L Fr | Na | Nd | Nd | Nd | Ns | 47 | 47 | Normal | Normal | Nd | Negative | Not detected | 0 |
| 68 | F | 23 | SPK: L CP | Na | Nd | Nd | Nd | Ns | 15 | 19 | Normal | Normal | Nd | Positive | + | 1 |
| 69 | M | 50 | SPK: L FC | Na | Nd | Nd | Nd | Ns | 46 | 46 | Normal | Normal | Nd | Positive | + | 1 |
| 70 | M | 17 | SPK: L FC | Na | Nd | Nd | Nd | Ns | 11 | 14 | Normal | Normal | Nd | Negative | Not detected | 0 |
| 71 | F | 22 | SW: Bil Fr (R>L) | Na | Nd | Nd | Nd | Ns | 14 | 18 | Normal | Normal | Nd | Negative | Not detected | 1* |
| 72 | M | 13 | SPK: R FC | L Fr | Nd | Nd | Nd | Ns | 7 | 10 | Normal | Normal | Nd | Negative | Not detected | 0 |
| 73 | M | 12 | SPK: R FC | Na | Nd | Nd | Nd | Ns | 8 | 9 | Normal | Normal | Nd | Positive | ++ | 1 |
| 74 | M | 18 | SPK: R FT | Na | Nd | Nd | Nd | Ns | 12 | 16 | Normal | Normal | Nd | Negative | Not detected | 0 |
| 75 | M | 13 | SPK: R FT | Na | Nd | Yes | Nd | Ns | 9 | 10 | Normal | Normal | Nd | Positive | Similar | 1 |
| 76 | M | 19 | SPK and SWC: R FCT | Na | Nd | Nd | Nd | Ns | 16 | 16 | Normal | Normal | Nd | Negative | Not detected | 0 |
| 77 | F | 13 | SPK: L TO | Na | Nd | Nd | Nd | Ns | 10 | 10 | Normal | Normal | Nd | QC (segmentation error) | Na | Na |
| 78 | M | 19 | SPK: L Fr | Na | Nd | Nd | Nd | Ns | 16 | 17 | Normal | Normal | Nd | QC (segmentation error) | Na | Na |
| 79 | F | 15 | SPK: R FT | Na | Nd | Nd | Nd | Ns | 11 | 13 | Normal | Normal | Nd | Negative | Not detected | 0 |
| 80 | F | 14 | SW: Bil FC (R>L), Ant Ver | R CP | Nd | Nd | Nd | Ns | 12 | 12 | Normal | Normal | Nd | Negative | Not detected | 0 |
| 81 | F | 25 | SPK: R TO, R TP | Na | Nd | Nd | Nd | Ns | 23 | 23 | Normal | Normal | Nd | Positive | ++ | 1 |
| 82 | F | 34 | SPK and SWC: R FT | R FT | Nd | Nd | Nd | Ns | 31 | 32 | Normal | Normal | Nd | Negative | Not detected | 0 |
| 83 | M | 21 | SW and SPK: L FT | Na | Nd | Nd | Nd | Ns | 12 | 20 | Normal | Normal | Nd | Negative | Not detected | 0 |
| 84 | M | 16 | SPK: L FT | Na | Nd | Nd | Nd | Ns | 14 | 15 | Normal | Normal | Nd | Negative | - | 0 |
| 85 | F | 10 | SPK: L FT | Na | Nd | Nd | Yes | ND FCD | 9 | 9 | Normal | Normal | Nd | Negative | Not detected | 0 |
| 86 | M | 11 | SPK: R PO | Na | Nd | Nd | Nd | Ns | 6 | 10 | Normal | Normal | Nd | Negative | Not detected | 0 |
| 87 | F | 19 | SPK: R CTP | Na | Nd | Nd | Nd | Ns | 15 | 17 | Normal | Normal | Nd | Positive | ++ | 2* |

**Supplementary Table 1.** Demographic and clinical characteristics of the enrolled participants. Abbreviations: Ant, anterior; Bi, bilateral; CP, centro-parietal; CTP, centro-temporo-parietal; F, female; Fr, frontal; FC, fronto-central; ND FCD, No definite FCD on histopathology (ILAE 2022[2]); FCP, fronto-centro-parietal; FCT, fronto-centro-temporal; FT, fronto-temporal; IFS, intermittent focal slow; L, left; M, male; Na, not available; Nd, not done; not detected, both 7T and 3T classifier detected no clusters; Ns, no surgery; O, occipital; OF, orbito-frontal; P, parietal; PO, parieto-occipital; QC, quality control exclusion due to movement artifacts in T1w and/or FLAIR MRI, MRI acquisition without whole-brain coverage, segmentation error, or abnormal feature values; R, right; Similar, similar clusters provided by 7T and 3T classifiers; SPK, spikes; SW, sharp waves; SWC, spike-wave complex; T, temporal; TO, temporo-occipital; TP, temporo-parietal; Ver, vertex; ++, 7T classifier outperforms 3T classifier; +, improved conspicuity of 7T classifier if compared with 3T classifier; -, 3T classifier outperforms 7T classifier; *, presence of false-positive clusters.

**References**

1.	Desikan RS, Ségonne F, Fischl B, et al (2006) An automated labeling system for subdividing the human cerebral cortex on MRI scans into gyral based regions of interest. Neuroimage 31:968–80. https://doi.org/10.1016/j.neuroimage.2006.01.021

2.	Najm I, Lal D, Alonso Vanegas M, et al (2022) The ILAE consensus classification of focal cortical dysplasia: An update proposed by an ad hoc task force of the ILAE diagnostic methods commission. Epilepsia 63:1899–1919. https://doi.org/10.1111/epi.17301

**Supplementary Fig. 1** 7T FLAIR images with superimposed cortical surfaces reconstructed from 7T T1w and FLAIR images. Coronal (**a**, Patient 27), sagittal (**b**, Patient 3) and axial (**c**, Patient 32) views. The *white* surface is in blue and the *pial* surface in red.

**Supplementary Fig. 2** Flowchart of patient selection and comparative diagnostic performance between 7T and 3T classifiers. The Eligible Patient cohort comprised 87 subjects, 17 of whom were excluded due to movement artefacts in T1w (6/17) or FLAIR MRI (3/17), no whole-brain 7T MRI acquisition (3/17), or surface reconstruction errors due to field inhomogeneities (5/17). The Final Patient cohort comprised 70 subjects (N = 70/87; 80.5%), whose results regarding lesion detection are detailed below. The flowchart on the left illustrates the subgroup of patients detected by 7T classifier (N = 40/70; 57.1%). Based on a direct comparison with the results of conventional 3T MRI, this subgroup is further subdivided into three distinct outcome categories: lesions detected exclusively by 7T classifier (N = 15/40; 37.5%), lesions showing improved conspicuity by 7T classifier than 3T classifier (N = 11/40; 27.5%), and lesions with similar performance between 7T and 3T classifier (N = 14/40; 35.0%). The right-hand flowchart shows patients with inconclusive 7T classification (N = 30/70; 42.9%), the majority of whom were not detected also by 3T classifier either (N = 25/30; 83.3%), and a smaller subset was exclusively detected by the 3T classifier (N = 5/30; 16.7%). Abbreviations: N, number of patients.

**Supplementary Fig. 3** Probability density profiles of *grey-white contrast* and *cortical thickness* features before and after ComBat harmonization. The plots display the distributions of the features highlighted in the saliency analysis, before (left column) and after (right column) ComBat harmonization, stratified by scanner strength (3T in blue, 7T in red) and left (LH) and right (RH) hemispheres. Profiles demonstrate that, before harmonization (left column), features patterns presented noticeable differences between the two field strengths. After ComBat harmonization (right column), we observed that the main distribution peaks for features at 3T and 7T largely overlapped, indicating successful shift of variations dependent on the reference scanner. After aligning the distribution centers for *grey-white contrast* feature (**a**-**d**), the 7T cohort maintained a higher and narrower peak density than the 3T distributions, reflecting the intrinsic higher tissue-contrast ratio of 7T MRI. For cortical thickness feature (**e**-**h**), the 7T (red) and 3T (blue) distributions overlapped after the initial leftward shift in the 7T data; however, 3T profile exhibited a higher peak density at the mode, as expected.

**Supplementary Fig. 4** Probability density profiles of sulcal depth feature before and after ComBat harmonization. The plots display the distributions of the features highlighted in the saliency analysis, before (left column) and after (right column) ComBat harmonization, stratified by scanner strength (3T in blue, 7T in red) and left (LH) and right (RH) hemispheres. Profiles demonstrate that, before harmonization (left column), features patterns presented noticeable differences between the two field strengths. After ComBat harmonization (right column), we observed that the main distribution peaks for features at 3T and 7T largely overlapped, indicating successful shift of variations dependent on the reference scanner. Following the application of ComBat, *sulcal depth* feature profiles (**a**-**d**) overlap effectively, demonstrating the successful mitigation of shifts dependent on the reference scanner to align with the training space of the MELD classifier.

**Supplementary Fig. 5** Probability density profiles of *intrinsic curvature* and *mean curvature* features before and after ComBat harmonization. The plots display the distributions of the features highlighted in the saliency analysis, before (left column) and after (right column) ComBat harmonization, stratified by scanner strength (3T in blue, 7T in red) and left (LH) and right (RH) hemispheres. The main peaks of the *intrinsic curvature* distributions (**a**-**d**) overlapped following harmonisation, the pronounced right-hand tail originally observed in the 7T profile remained unchanged, suggesting that the higher spatial resolution of 7T MRI may allow for a better characterisation of localised variations in curvature. Following the application of ComBat, the primary modes of the distributions of *mean curvature* feature (**e**-**h**) overlap effectively, demonstrating the successful mitigation of shifts dependent on the reference scanner to align with the training space of the MELD classifier.

**Supplementary Fig. 6** Probability density profiles of *white-matter FLAIR intensity* and *grey-matter FLAIR intensity* features before and after ComBat harmonization. The plots display the distributions of the features highlighted in the saliency analysis, before (left column) and after (right column) ComBat harmonization, stratified by scanner strength (3T in blue, 7T in red) and left (LH) and right (RH) hemispheres. Following the application of ComBat, the primary modes of the distributions of *white-matter FLAIR intensity* (**a**-**d**) and *grey-matter FLAIR intensity* (**e**-**h**) features overlap effectively, demonstrating the successful mitigation of shifts dependent on the reference scanner to align with the training space of the MELD classifier. Despite the alignment of the central peaks, we observed that the post-harmonization profiles reveal that certain distribution shapes retain their original characteristics as a function of field strength, confirming that the variance at 7T is preserved.
